# Mating-Induced Differential Expression in Genes Related to Reproduction and Immunity in *Spodoptera litura* (Lepidoptera: Noctuidae) Female Moths

**DOI:** 10.1093/jisesa/ieaa003

**Published:** 2020-02-24

**Authors:** Bo Gao, Xiao-Qian Song, Hong Yu, Da-Ying Fu, Jin Xu, Hui Ye

**Affiliations:** 1 School of Life Sciences, Yunnan University, Kunming, China; 2 Yunnan Academy of Biodiversity, Southwest Forestry University, Kunming, China

**Keywords:** *Spodoptera litura*, RNAseq, mating-responsive gene, immunity, reproduction

## Abstract

Mating promotes reproductive activity, which may impact immune performance. Paradoxically, mating frequently challenges females’ immunity (e.g., infections). Therefore, studies of postmating resource allocation between reproduction and survival are likely to shed new light on life-history trade-off and sexual selection. Here, we used RNAseq to test whether and how mating affected mRNA expression in genes related to reproduction and immunity in *Spodoptera litura* female moths. Results show a divergent change in the differentially expressed genes (DEGs) between reproduction and immunity: the immune response was largely downregulated shortly after mating (~6 h postmating), which has some recovery at 24 h postmating; reproductive response is trivial shortly after mating (~6 h postmating), but it largely upregulated at 24 h postmating (e.g., egg maturation related genes were highly upregulated). Considering the fact that most of the total DEGs downregulated from 0 to 6 h postmating (from 51/68 to 214/260) but most of the total DEGs upregulated at 24 h postmating (816/928), it is possible that trade-offs between reproduction and immunity occurred in mated females. For example, they may shut down immunity to favor sperm storage and save limited resources to support the increased energy required in reproduction (e.g., egg maturation and oviposition). Mating-induced infections should be trivial due to low polyandry in *S. litura*. A reduced immune defense may have no threat to *S. litura* survival but may benefit reproduction significantly. Furthermore, obvious expression changes were detected in genes related to hormone production, suggesting that endocrine changes could play important roles in postmating responses.

A number of studies have suggested that mating positively affects female reproduction activity but may negatively affect female immune performance in insects (reviewed in Schwenke et al. 2016). However, a recent review by Oku et al. (2019) has pointed out that the evidence is still insufficient to fully answer the hypothesis ‘*does mating negatively affect female immune defences in insects?*’ In the 22 studies involving 15 insect species reviewed by Oku et al. (2019), mating upregulates female immune responses in some species, whereas it downregulates female immune responses in some others, and in some insects there is no difference in immune responses between mated and virgin females and likewise, measures of different immune indicators may result in reverse consequences in the same species. These diverse findings may result from different mating systems. For instance, polygamous mating systems can generate intense postmating sexual conflict (Dawkins 1976), which can cause high costs in females and thus may affect postmating immune response. On the one hand, males may suppress female immunity directly to promote sperm storage in female reproductive tracts and egg fertilization by their sperm (Wigby et al. 2019). On the other hand, mating can challenge the female’s immune system by transferring foreign materials and infections to females (Smith and Dobson 1992, Whitlow 2004, Okada et al. 2017, Oku et al. 2019); females of polyandrous species should have higher postmating immunity if sexually transmitted infection is the major factor driving female postmating costs (Oku et al. 2019). Therefore, studies of mating-induced regulation on reproduction and immunity in different mating systems will provide evolutionarily insights into reproductive strategies and sexual selection in females.

Mating is an essential behavioral and physiological process for sexual reproduction in insects. It is obvious that mating can cause major changes in the physiology and behavior in females. However, the linkage (i.e., the regulation process and molecular mechanisms) between mating and postmating physiological and behavioral changes in females is still a mystery in most insects. Studies have investigated the effects of mating on female gene expression in a number of insect species and found mating-induced expression changes in many (dozens to hundreds) genes, such as transcription factors, metabolic enzymes, and genes related to hormones synthesis, immune defense (e.g., antimicrobial peptide genes), egg maturation (e.g., *yolk protein*, *chorion protein*), nutrient distribution, behavior, and aging (e.g., Dalton et al. 2010, Bono et al. 2011, Immonen and Ritchie 2012, Zhou et al. 2014). Moreover, studies also demonstrated that reproduction-related physiological changes are often under endocrine control (e.g., juvenile hormone, ecdysone), and these hormones are also involved in immune response (Schwenke et al. 2016, Schwenke and Lazzaro 2017). The balance between juvenile hormone (JH) and 20-Hydroxyecdysone (20E) is important for activation of egg maturation, with increased JH levels upregulating the expression of vitellogenin or yolk protein genes, promoting the uptake of vitellogenin or yolk protein into oocytes and aiding in the progression of developing follicles (Schwenke et al. 2016). JH and 20E also have opposite effects on immune defense in many insects. For example, JH reduces phenoloxidase (which plays an important role in immune defense) activity in the beetle *Tenebrio molitor* (Rolff and Siva-Jothy 2002) and reduces the expression of antimicrobial peptide genes in the fruit fly *Drosophila melanogaster* (Flatt et al. 2008). In contrast, 20E promotes the expression of antimicrobial peptide genes in *D. melanogaster* (Meister and Lagueux 2003, Flatt et al. 2008, Zhang and Palli 2009). Such opposite effects of JH and 20E on reproduction and immunity suggest the possibility that the levels of the two hormones may mediate a trade-off between reproduction and immunity. In addition, both reproduction and immunity are also responsive to insulin, with elevated insulin promoting oogenesis but inhibiting immune responses (Schwenke et al. 2016). The effect of insulin signaling on reproduction and immunity may not be independent of JH and 20E, which may partially act through JH and 20E (Schwenke et al. 2016). Studies on mating-induced endocrine and gene expression changes are helpful to clarify gene functions and the connection between hormones and gene expression (Mack et al. 2006).

Lepidopterans usually do not feed on a protein source as adults; instead, they sequester most of the protein needed for egg production and basal maintenance during their larval stage (Gilbert 1972, Baker and Baker 1973). Therefore, in many Lepidoptera species, including *Spodoptera litura* (Yu et al. 2014, Xu et al. 2018), both males and females have a limited protein supply (Gilbert 1972, Baker and Baker 1973). Males and females, therefore, may have evolved resource allocation strategies between reproduction and survival, particularly in polygamous species (Li et al. 2014c, Xu and Wang 2014, Yu et al. 2014, Xu et al. 2019a). Sexual conflict was thus raised as the reproductive interests are different and often antagonistic between the sexes (Dawkins 1976). Therefore, lepidopterans can be good models for the study of mating-induced regulation in reproduction and immunity under such antagonistic circumstances. In addition, lepidopterans also are important model systems for innate immunity of insects (Jiang et al. 2010). Many caterpillars are agricultural pests and thus understanding their immune systems has potential practical significance.

The common cutworm moth, *Spodoptera litura* is a major agricultural pest worldwide due to its polyphagy, gluttony, alternating generations, and strong pesticide resistance (Armes et al. 1997, Zhou and Huang 2002). The draft genome sequences of *S. litura* have been published recently (Cheng et al. 2017). This insect is a nocturnal moth in which all adult reproductive activities (calling, courtship, mating, and oviposition) take place during the night (Li et al. 2012). Adults eclose at dusk, but no mating takes place on the night of emergence and maximum mating (about 70%) occurs during the second night after emergence, and those unmated will mate during the third night (Li et al. 2012). Our previous studies have found that matings or male accessory gland (MAG) secretions induce significant changes in female reproductive physiology and behavior (Li et al. 2012, Li et al. 2014a, Yu et al. 2014, Xu et al. 2019b). MAG secretions not only trigger oviposition but also promote egg development (Yu et al. 2014). MAG secretions also show negative effects on female longevity, which may be because MAG secretions stimulate females to allocate more resources to egg development and oviposition, leaving fewer resources for adult survival (Yu et al. 2014). Most females start to lay fertilized eggs on the subsequent night after mating (Li et al. 2012).

Based on the above findings in *S. litura* and other insect species, we hypothesize that 1) the postmating behavioral and physiological changes in females is related to gene expression, 2) mating will positively affect the activity of reproductive-related genes but negatively affect the activity of immunity-related genes in females. To test these hypotheses, we performed transcriptome differential analysis between virgin and mated females at three time points (0, 6, and 24 h postmating). The previous substantial study on reproductive behavior and physiology in *S. litura* (see references above) and the reliable sequencing data and in-depth gene mining in this study allow us to discuss the evolutionary significance of the postmating gene expression regulation between reproduction and immunity.

## Materials and Methods

### Insect Rearing and Sample Collection

*S. litura* larvae were reared on an artificial diet (Li et al. 1998) at 25 ± 1°C and a relative humidity of 60–70% with a photoperiod of L14:D10. Pupae were collected from the colony and sexed according to the morphology of exterior paramera (Li et al. 2006). Male and female pupae were maintained in separate cages to ensure virginity of adults after eclosion. In order to minimize the effect of age variance, newly eclosed moths, eclosed during 1 h before lights off to 1 h after lights off (eclosion peaked during these 2 h; Li et al. 2014b), were collected and reared for subsequent mating experiments. Male and female moths were reared in separate cages under the same environmental conditions and fed with 10% honey solution.

The mating duration of this insect is about 40 min (Li et al. 2012). Matings were allowed by pairing 1-d-old virgin moths during 4–5 h (only those females that started to mate during this hour were collected after mating for subsequent RNAseq) after lights off in the second scotophase after eclosion (about 28 h after eclosion), with one pair per box. Mating events (two insects engaged at the tip of the abdomen) were recorded (Li et al. 2012). The mated females were then individually caged and their whole bodies were sampled at 0 h (immediately after mating), 6 and 24 h after mating. Seven females were used as a replicate, and two replicates were used for each sampling time point. Matings were verified by dissecting the females to check for the presence of a spermatophore in the mating sac (Li et al. 2012). The spermatophore was then removed from the bursa. Virgin females at the same age as mated individuals were used as controls. All samples were placed in the liquid nitrogen for immediate freezing after sampling and stored at −80°C.

### cDNA Library Preparation and Sequencing

Total RNA was extracted from samples using Trizol reagent (Invitrogen, USA) and the concentration and purity of RNA were measured by using Qubit RNA Assay Kit (Life Technologies, USA) and the NanoPhotemeter spectrophotometer (IMPLEN, USA). The integrity of RNA was detected by the RNA Nano 6000 Assay Kit (Agilent Technologies, USA). A total of 3 μg RNA per sample was used for the preparation of the sequencing libraries by using NEBnext Ultra RNA Library Prep Kit for Illumina (NEB, USA) following the manufacturer’s instructions and index codes were added to attribute sequences to each sample.

The clustering of the index-coded samples was performed on a cBot Cluster Generation System using TruSeq PE Cluster Kit v3-cBot-HS (Illumina, San Diego, CA) according to the manufacturer’s protocol. After cluster generation, the library preparations were sequenced on an Illumina HiseqTM 4000 platform and 125 bp/150 bp paired end reads were generated.

### Quality Control and Assembly

The original data was filtered to ensure the quality and reliability for further analysis, which mainly includes removing the reads of the adapter, reads containing N (N means that the base information cannot be determined) and low-quality reads (the number of bases with Qphred ≤ 20 accounts for more than 50% of the total read length) from the raw data. The values of Q20, Q30, and GC content of the clean data were calculated. These clean reads were then mapped to the reference genome sequence of *S. litura* (https://www.ncbi.nlm.nih.gov/genome/?term=spodoptera+litura) using Hisat2 v2.0.5 software.

### Differential Expression Analysis

Gene expression levels were analyzed by using the expected number of Fragments Per Kilobase of transcript sequence per Millions base pairs sequenced (FPKM) method. The differential expression analysis between samples was performed using the edgeR R package (3.0.8). *P*-value was adjusted using *q*-value (Storey 2003). *q* < 0.05 and |log2(foldchange)|>1 was set as the threshold for significantly differential expression.

### Functional Annotation and Enrichment Analysis of Differentially Expressed Genes

Using the BLAST (Altschul et al. 1997) software, the differentially expressed genes (DEGs) were compared with NCBI non-redundant protein (NR) and nucleotide sequence (NT), Swiss-Prot protein database (Swiss-Prot), Gene Ontology (GO), KEGG Orthology (KO), Cluster of Orthologous Groups (COG), Clusters of EuKaryotic Orthologous Groups (KOG), and Pfam databases to obtain annotation information about the DEGs. GO enrichment analysis of DEGs was implemented by using the GOSeq program and KEGG enrichment was performed using the KOBAS software. GO terms and KEGG pathways with *q* < 0.05 were significantly enriched in DEGs.

### Validation by qRT-PCR

The total RNA of females was extracted using RNAiso plus (TaKaRa, China), and the cDNA synthesis was performed using PrimeScript RT reagent Kit with gDNA Eraser (Perfect Real Time) (Takara, China). The qRT-PCR primers were designed by Prime Premier 6.0 (Supp Table S1 [online only]). Real-time quantitative PCR was performed with QuantStudio 7 Flex (Thermo Fisher Scientific, USA) using the following program: 95°C for 30 s, followed by 40 cycles of 95°C for 5 s, 60°C for 30 s and dissociation. *Actin* (GeneBank ID: 111359844) was used as a reference gene (Teng et al. 2012, Lu et al. 2015, Xu et al. 2019b). The 2^-ΔΔCT^ method (Livak and Schmittgen 2001) was used to calculate the relative expression. Differences of gene expression levels between treatments were analyzed by one-way ANOVA followed by LSD test for multiple comparisons. All analyses were conducted using SPSS 22.0. The rejection level was set at α<0.05. All values are reported as mean ± SE.

## Results

### Sequencing and Assembly

By RNAseq using Illumina HiSeq4000 platform, ~60,000,000 clean reads were obtained from each of the 12 sequenced libraries (Supp Table S2 [online only]). The percentages of Q20 and Q30 of all samples’ clean reads ranged from 96.46% to 97.59% and from 91.09% to 93.46%, respectively. The mapped ratios of all samples’ clean reads to the reference genome sequences ranged from 88.29% to 91.35%. The results also showed that approximately 48% of the total number of genes were expressed (1 ≤ RPKM ≤ 60), and approximately 7% were highly expressed (RPKM > 60) in all groups (Supp Table S3 [online only]). The biological replicates were highly correlated (Supp Table S4 [online only]), which affirmed the technical reproducibility of the RNASeq technology and reproducibility of biological replicates. The transcriptome raw reads have been deposited with the NCBI SRA database (accession no.: SRR10023496~SRR10023507).

### Overview of Mating-Induced Transcriptional Changes

**Fig. 1.**
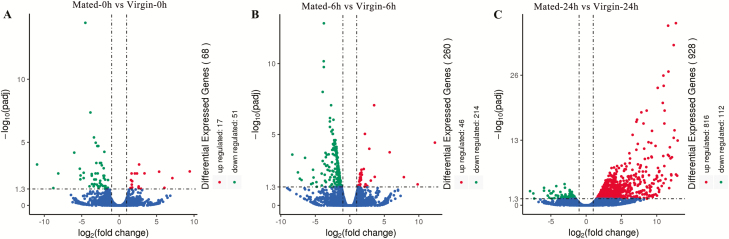
Volcano plots of the DEGs in Mated-0h versus Virgin-0h group (A), Mated-6h versus Virgin-6h group (B), and Mated-24h versus Virgin-24h group (C). Genes with significant differential expression were indicated by red dots (upregulated) and green dots (downregulated). Genes with no significant differential expression were represented by blue dots.

**Fig. 2.**
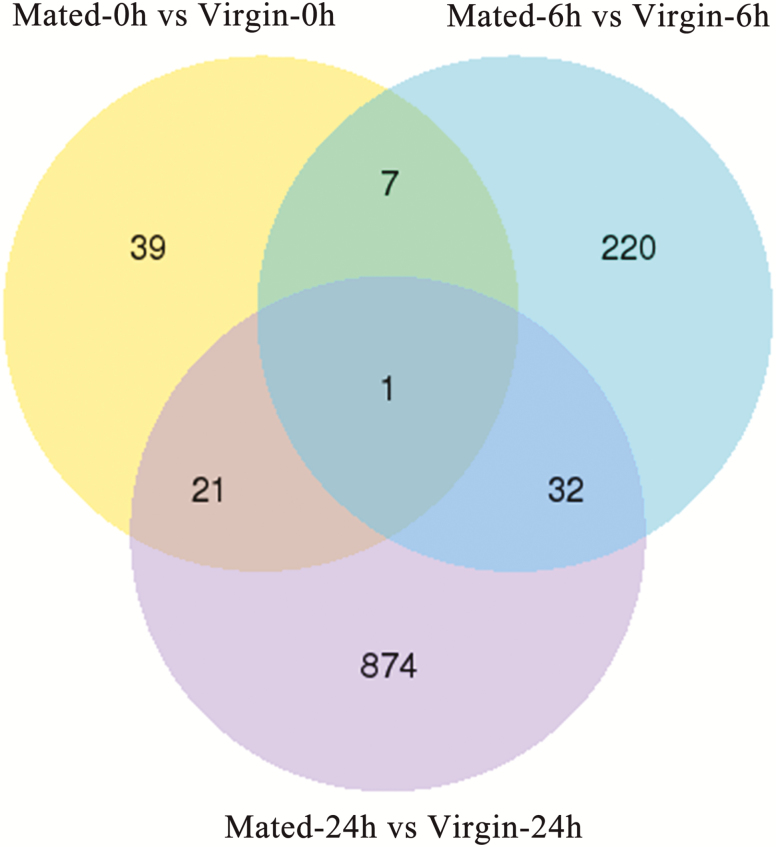
The Venn diagram of DEGs. Yellow circle indicates the number of DEGs in Mated-0h versus Virgin-0h group, light blue circle indicates the number of DEGs in Mated-6h versus Virgin-6h group, and purple circle indicates the number of DEGs in Mated-24h versus Virgin-24h group. The overlapping circles represented common DEGs among all combinations.

There are 68, 260, and 928 DEGs within Mated-0h versus Virgin-0h, Mated-6h versus Virgin-6h and Mated-24h versus Virgin-24h groups, respectively (Fig. 1). ‘Novel’ started gene ID means this gene is new relative to the previously published genome sequence of *S. litura*. There were eight genes shared between Mated-0h versus Virgin-0h DEGs and Mated-6h versus Virgin-6h DEGs, 21 genes shared between Mated-0h versus Virgin-0h DEGs and Mated-24h versus Virgin-24h DEGs, and 33 genes shared between Mated-6h versus Virgin-6h DEGs and Mated-24h versus Virgin-24h DEGs, while only one gene (*Novel00477*, *cecropin*; all downregulated) was shared by the three DEGs groups (Mated-0h versus Virgin-0h DEGs, Mated-6h versus Virgin-6h DEGs and Mated-24h versus Virgin-24h DEGs) ((Fig. 2); Supp Table S5 [online only]).

To better understand their functions, particularly those novel genes, all these DEGs were annotated based on NR, NT, Swiss-Prot, GO, KO, COG, KOG, and Pfam databases (Supp Tables S6, S9, and S12 [online only]). These DEGs were then mapped to the terms in the GO database. Each of 45 GO terms were enriched in Mated-0h versus Virgin-0h, Mated-6h versus Virgin-6h and Mated-24h versus Virgin-24h groups, respectively (Supp Fig. S1 [online only]; Supp Tables S7, S10, and S13 [online only]). DEGs were also mapped to KEGG pathways for analysis. The top 20 pathways of each group by the KEGG enrichment analysis were shown in Supp Fig. S2 [online only] and Supp Tables S8, S11, and S14 [online only].

Based on above annotation and enrichment analysis, we further analyzed mating-induced gene expression regulation pattern in relation to reproduction and immunity, and the results are presented in Tables 1 and 2 and described below.

**Table 1.** Mating-induced expression changes in genes related to reproduction in *S. litura* females

GeneID	log2FoldChange	padj	Annotation	Function	Reference
Mated-0h versus Virgin-0h					
*111358169*	−1.8720	0.009102	Dopamine receptor-interacting protein	Dopamine inhibits JH degradation in young but stimulates it in mature females.	Gruntenko et al. 2005
*111355246*	−3.3447	0.021946	Odorant receptor family	Oviposition and/or host location by females.	Leal 2013, Venthur and Zhou 2018
*111350604*	−10.9643	0.000574	Yolk protein-like	Yolk formation.	LeMotte et al. 1989, Van Rompay et al. 2015, Upadhyay et al. 2016
Mated-6h versus Virgin-6h					
*111361107*	1.4395	0.037998	Insulin-degrading enzyme-like	Elevated insulin promotes oogenesis and inhibits immune responses.	Galagovsky et al. 2014, Schwenke et al. 2016
*111364657*	1.3698	0.020863	Structural constituent of chorion	Plays vital role in oocytes and embryo development in insects.	LeMotte et al. 1989, Van Rompay et al. 2015, Upadhyay et al. 2016
*111348993*	−1.6956	0.003881			
*111352474*	−1.3772	0.021956	Odorant receptor family	Oviposition and/or host location by females.	Leal 2013, Venthur and Zhou 2018
*111364510*	−2.3686	9.14E-07	Ecdysteroid UDP-glucosyltransferase	Positive modulator of fecundity.	Ge et al. 2019
Mated-24h versus Virgin-24h					
Pheromone binding					
*Novel00212*	12.031	1.02E-05			
*Novel00717*	8.6144	0.048854			
*Novel00577*	3.1205	0.003843	Pheromone-binding protein family	Bind and transport the sex pheromones.	Liu et al. 2013
*Novel00589*	2.0660	0.003787			
*Novel00402*	1.8146	0.028146			
Odorant binding					
*111355246*	7.1458	0.030157			
*111357535*	3.981	0.002073			
*111355452*	3.4107	0.00301			
*111357248*	1.6643	0.0384			
*111348794*	−1.9696	0.006937	Odorant receptor family	Oviposition and/or host location by females.	Leal 2013, Venthur and Zhou 2018
*111356886*	−2.3014	0.010417			
*111348468*	−4.8306	0.045387			
*111356816*	−4.8769	0.003944			
*111356783*	−7.3552	0.045404			
Egg development					
*111350604*	8.0320	1.83E-07	Yolk protein-like	Plays vital role in oocytes and embryo development in insects.	LeMotte et al. 1989, Van Rompay et al. 2015, Upadhyay et al. 2016
*111363907*	2.2214	0.009967	Vitellogenin-like		
*111350990*	7.2973	5.39E-06	Vitellogenin receptor isoform X1		
*111356264*	12.7292	7.09E-07	Chorion peroxidase		
*111356257*	6.5819	0.00395525	Sex combs reduced-like		
Insulin					
*111352888*	3.5731	1.22E-06	Insulin-like growth factor-binding protein	Elevated insulin promotes oogenesis and inhibits immune responses.	Galagovsky et al. 2014, Schwenke et al. 2016
*111360216*	1.9168	0.029822	Insulin-related hormone activity		
*111355766*	−3.9729	0.036033	Bombyxin family; Insulin-related peptide of insects		
*111349683*	−5.9624	0.000325			
*111358813*	−7.9472	0.001517			
Juvenile hormone related					
*111350212*	5.2992	0.000363	Juvenile hormone esterase-like	Juvenile hormone degradation.	Audsley et al. 2008, Pietrzyk et al. 2013
*111358473*	3.4352	1.05E-05			
*111349341*	2.6958	3.41E-05			
*111350611*	2.3905	0.004151			
*111364670*	4.6005	0.008158	Juvenile hormone epoxide hydrolase-like		
*111357658*	3.4065	0.008319			
*111353831*	4.3478	1.83E-07	Juvenile hormone diol kinase		
*111357756*	3.8761	5.06E-05	Juvenile hormone-binding protein-like	Protect the labile hormone molecules from degradation by esterases.	
*Novel00777*	2.3398	0.000977			
Ecdysone related					
*111359732*	5.6206	6.45E-08	Ecdysone oxidase	Function in ecdysone synthesis.	Wang et al. 2018
*Novel00281*	−1.6035	0.047915	Ecdysone oxidase		
*111361821*	4.4706	0.007314	Ecdysone 20-monooxygenase	Catalyzes the ecdysone reaction.	Rauschenbach et al. 2008
*111360733*	3.5502	0.000278	Ecdysone-induced protein 78C-like		
Ecdysteroid related					
*111355824*	6.2422	0.021351			
*111348884*	3.8355	1.44E-05			
*111355746*	3.6249	8.60E-06			
*111349048*	3.5978	0.008271	Ecdysteroid UDP-glucosyltransferase	Positive modulator of fecundity.	Ge et al. 2019
*111355676*	3.5567	0.008087			
*111348860*	2.8236	0.045152			
*111348883*	2.1452	0.022315			
*111364512*	2.1406	0.000850			
Hormone related					
*111351503*	11.2527	5.79E-06	Octopamine receptor 1-like	Octopamine inhibits JH degradation both in young and mature *Drosophila* females.	Gruntenko et al. 2005
*111358169*	−1.8720	1.86E-10	Dopamine receptor-interacting protein	Dopamine inhibits JH degradation in young but stimulates it in mature females.	Gruntenko et al. 2005
Pheromone production					
*111354673*	7.3795	3.56E-06	Alcohol-forming fatty acyl-CoA reductase	Catalyze reduction of a fatty acyl‐CoA to the corresponding alcohol in insect pheromone biosynthesis. Play an important role in determining the proportion of each component in the pheromone blend.	Finet et al. 2019
*111354886*	5.7090	1.73E-08			
*111354889*	5.1114	0.002344			
*111354723*	4.5612	0.003722			
*111356581*	4.2320	0.000116			
*111354725*	3.8179	0.008468			
*111354672*	3.6328	0.001969			

**Table 2.** Mating-induced expression changes in genes related to immunity in *S. litura* females

GeneID	log2FoldChange	padj	Annotation	Function	Reference
Mated-0h versus Virgin-0h					
Antimicrobial peptides					
*Novel00496*	−1.82271	0.029622	Cecropin family	Insect antimicrobial peptides.	Wu et al. 2018
*Novel00495*	−2.55838	0.000435			
*Novel00477*	−2.57121	0.017087			
*111357054*	−3.11401	0.008898			
*111363355*	−1.99107	0.004434	Lebocin family		
Mated-6h versus Virgin-6h					
Lysozyme					
*111348461*	−2.98959	0.001993	Lysozyme	Defends against bacterial infection by hydrolyzing the bacterial cell walls and causing bacterial lysis.	Ragan et al. 2009
Antimicrobial peptides					
*111357147*	−3.21448	0.003131	Attacin family	Insect antimicrobial peptides.	Wu et al. 2018
*111357146*	−3.33851	0.000267			
*111357148*	−3.42633	0.001637			
*111364705*	−3.63371	0.006909			
*111364839*	−3.66288	0.001202			
*Novel00587*	−2.15271	0.009609	Cecropin family		
*Novel00495*	−2.2755	0.010099			
*Novel00477*	−2.3701	0.004032			
*Novel00496*	−2.58578	0.032345			
*111357054*	−3.05654	0.000251			
*111357274*	−2.64676	0.035106	Defense protein 4		
*111364668*	−3.52146	0.000198	Gloverin family		
*111364829*	−4.05679	0.039562			
*111363355*	−2.59232	0.00684	Lebocin family		
*Novel00512*	3.522017	8.63E-08	Moricin family		
*111357548*	3.043572	0.017847	Moricin family		
Mated-24h versus Virgin-24h					
Lysozyme					
*111362447*	5.978958	0.011857	Lysozyme	Defends against bacterial infection by hydrolyzing the bacterial cell walls and causing bacterial lysis.	Ragan et al. 2009
*111364585*	2.321828	0.013781			
Phenoloxidase					
*111360037*	3.485551	0.00226	Phenoloxidase subunit 1	Phenoloxidase involved in defensive melanization and production of oxidative free radicals.	Schwenke et al. 2016
*111359934*	1.922081	0.038305	Phenoloxidase subunit 2		
Fungal protease inhibitor					
*111352156*	2.663437	7.87E-05	Fungal protease inhibitor	Against fungal infection.	Roy et al. 2009
Antimicrobial peptides					
*111364839*	−4.64024	0.001715	Attacin family	Insect antimicrobial peptides.	Wu et al. 2018
*111364705*	−5.96934	0.001232			
*111351795*	8.303923	0.005662	Cecropin family		
*Novel00710*	6.280496	0.005348			
*111357132*	4.629945	0.018206			
*Novel00251*	4.444034	0.005489			
*Novel00706*	4.158364	0.042942			
*111349620*	3.946093	0.001683			
*111357351*	2.525735	0.000239			
*Novel00477*	−2.9696	0.046894			
*111355308*	−3.05549	5.85E-05			
*111364668*	−4.50589	0.037374	Gloverin family		
*111364829*	−4.86408	0.048854			
*111357274*	−5.22352	0.001272	Defense protein 4		

### Transcriptional Changes During Mating (0 h Postmating)

Within the 68 DEGs between Mated-0h and Virgin-0h groups, 51 genes were downregulated and 17 genes were upregulated in mated females at the time of 0 h postmating compared to virgin females (Fig. 1A). The log2FoldChange (LFC) value of DEGs varied from −10.96 to 9.43 (Supp Table S6 [online only]).

Only three reproductive-related genes were found within these DEGs (Table 1). They are *111358169* (*Dopamine receptor-interacting protein*), 111355246 (*Odorant receptor*), and *111350604* (*Yolk protein*), which may function in egg development and oviposition and/or host location. However, all three genes were downregulated in mated females at the time of 0 h postmating compared to virgin females.

Similarly, only five immunity-related genes were found within these DEGs (Table 2). They all encode antimicrobial peptides, with four of them belonging to the Cecropin family and one belonging to the Lebocin family. All of these antimicrobial peptides were also downregulated in mated females at 0 h postmating compared to virgin females.

### Transcriptional Changes at 6 h After Mating

At 6 h after mating, 260 DEGs were identified between mated and virgin groups, in which 214 genes were downregulated and 46 genes were upregulated in mated females at the time of 6 h postmating compared to virgin females (Fig. 1B). The LFC value of DEGs differed from −8.33 to 12.33 (Supp Table S9 [online only]).

Still fewer (five) reproductive related genes were found within these DEGs with two upregulated and three downregulated (LFC ranged from −2.36 to 1.43) (Table 1). The two upregulated genes were *111361107* (*Insulin-degrading enzyme*), *111364657* (*Structural constituent of chorion*). The upregulation of *111364657* may promote oogenesis, while the upregulation of *111361107* may inhibit oogenesis. The three downregulated genes include *111348993* (*Structural constituent of chorion*), *111352474* (*Odorant receptor*), and *111364510* (*Ecdysteroid UDP-glucosyltransferase*; a positive modulator of fecundity).

Relative more (17) immunity-related genes were found within these DEGs. One is *Lysozyme* (*111348461*; downregulated) and others were antimicrobial peptides (2 upregulated and 14 downregulated) (Table 2). Among the antimicrobial peptides, five belong to the Attacin family (LFC: −3.66 to −3.21), five belong to the Cecropin family (LFC: −3.05 to −2.15), two belong to the Gloverin family (LFC: −4.05 to −3.52), two belong to the Moricin family (LFC: 3.04 to 3.52), one belong to the Lebocin family (LFC: −2.59) and one is Defense protein 4 (LFC: −2.64).

### Transcriptional Changes at 24 h After Mating

There were 928 DEGs between Mated-24h and Virgin-24h groups, of which 112 genes were downregulated and 816 genes were upregulated in mated females at the time of 24 h postmating compared to virgin females (Fig. 1C). The LFC value of DEGs changed from −7.45 to 12.96 (Supp Table S12 [online only]).

A total of 54 reproductive related genes were found within the 928 DEGs with 10 of them downregulated and 44 upregulated (Table 1). These genes can be divided into four categories: 1) hormone related, including five Insulin-related genes (two upregulated and three downregulated), nine JH related genes (all upregulated), four Ecdysone related genes (one downregulated and three upregulated), eight *Ecdysteroid UDP-glucosyltransferases* (all upregulated), one *Octopamine receptor* (upregulated) and one *Dopamine receptor-interacting protein* (upregulated); 2) egg development related, including *Yolk protein*, *Vitellogenin* and its receptor, *Chorion peroxidase* and *Sex combs reduced*, all upregulated with LFC ranged from 2.22 to 12.72; 3) olfactory activity related, including five *Pheromone-binding proteins* (all upregulated) and nine *Odorant receptors* (four upregulated and five downregulated); 4) pheromone production related, all of the seven genes were *Alcohol-forming fatty acyl-CoA reductase* (all upregulated).

Relatively fewer (19) immunity-related genes were found within these DEGs (Table 2), including two *Lysozyme* (LFC: 2.32 to 5.97), two *Phenoloxidase* (LFC: 1.92 to 3.48), one *Fungal protease inhibitor* (LFC: 2.66) and 14 Antimicrobial peptides (seven upregulated and seven downregulated; LFC: −5.96 to 8.30).

### Validation of RNAseq Results by qRT-PCR

**Fig. 3.**
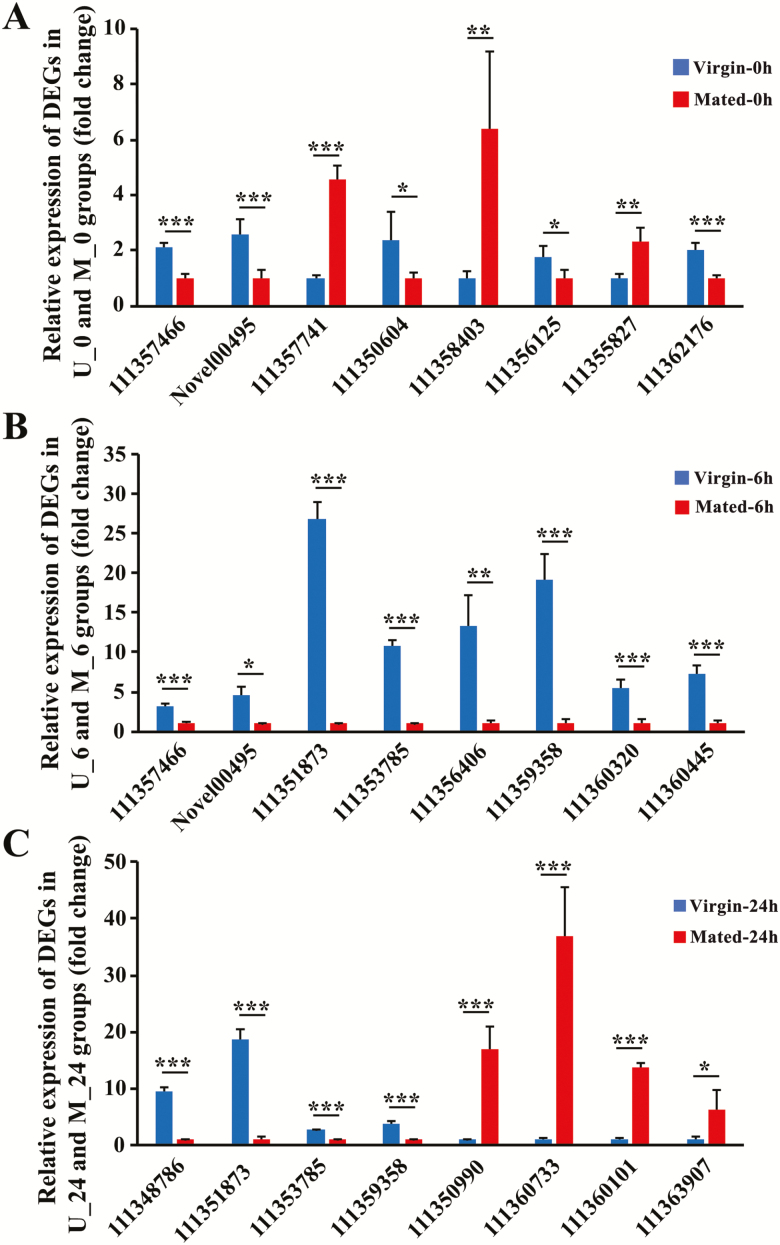
The transcriptome validation for DEGs by quantitative real-time PCR. (A) Relative expression of DEGs from Mated-0h versus Virgin-0h group, (B) Relative expression of DEGs from Mated-6h versus Virgin-6h group, and (C) Relative expression of DEGs from Mated-24h versus Virgin-24h group. *P < 0.05; **P < 0.01, ***P < 0.001. Error bars indicate SE.

To verify the accuracy of the Illumina sequencing data, qRT-PCR was performed on 24 DEGs (eight genes each from the Mated-0h vs Virgin-0h, Mated-6h vs Virgin-6h and Mated-24h vs Virgin-24h groups, respectively), which includes four reproduction-related genes (*111350604*, *111350990*, *111360733* and *111363907*) and one immunity-related gene (*Novel00495*) (Fig. 3). The expression levels of these genes measured by qRT-PCR were similar to the results from the RNAseq analysis. These results suggest that the RNAseq data were reliable.

## Discussion

In the present study, we showed that the number of DEGs (with twofold or greater changes and *q* < 0.05) was small (68) immediately after mating, which then increased to 260 at 6 h postmating and then soared to 928 at 24 h postmating (Fig. 1). *S. litura* is a nocturnal moth and all reproductive activities take place during the night (Li et al. 2012). After mating, *S. litura* females showed reduced activity in the remaining nighttime hours after mating and during the subsequent daytime hours the next day, after which they will start to lay eggs during the subsequent night following mating (about 20 h postmating) and showed high levels of oviposition-site searching activity (Li et al. 2012). Therefore, the pattern of gene expression changes after mating (Fig. 1) is consistent with the postmating behavioral and physiological changes reported earlier in *S. litura* females (Li et al. 2012). Similar change patterns of gene expression after mating also have been shown in the reproductive tract of the mosquito *A. aegypti* (the no. of DEGs increased from 76 to 290 during 0–24 h postmating) (Alfonso-Parra et al. 2016) and the head of *D. melanogaster* (the no. of DEGs increased from 237 to 545 during 0–72 h postmating) (Dalton et al. 2010), while a reverse pattern was shown in the whole body of *D. melanogaster* (the no. of DEGs decreased from 64 to 10 during 1–24 h postmating) (McGraw et al. 2008). In the whole body of the mosquito *Anopheles gambiae*, the number of DEGs increased from 14 to 65 during 2–6 h postmating but it decreased to 40 at 24 h postmating (Rogers et al. 2008). In female moths of *Ostrinia nubilalis*, the number of DEGs in bursa copulatrix is 345 and in bursal gland is 633 at 0 h postmating (Al-Wathiqui et al. 2016). Although the variability and sensitivity of the above studies may be affected by different methods and different laboratory work, these results suggest that the mating-induced gene expression changes can vary greatly between different species and tissues. Nevertheless, the number of mating-induced DEGs were still very small in comparison to the whole genome in *S. litura* (Cheng et al. 2017) and other species, which further supports the hypothesis that sexually mature females are molecularly ‘poised’ to respond to mating (Heifetz and Wolfner 2004, McGraw et al. 2004, Mack et al. 2006).

Insect immune defenses are carried out through humoral and hemocyte responses. The humoral response occurs by synthesis of antimicrobial peptides, while hemocytes play defensive roles through encapsulation and phagocytosis (Jiang et al. 2010). Antimicrobial peptides are synthesized by hemocytes or the fat body and then are secreted into the hemolymph to kill microbes. More than 500 different antimicrobial peptides have been reported in insects (Theis and Stahl 2004, Wu et al. 2018). In the present study, most of the immunity-related DEGs (Table 2) encoded antimicrobial peptides and a few genes related to lysozyme and phenoloxidase synthesis. Lysozyme can hydrolyze the bacterial cell walls and cause bacterial lysis, whereas phenoloxidase is involved in defensive melanization and production of oxidative free radicals (Ragan et al. 2009, Schwenke et al. 2016).

Compared to immunity-related DEGs, the categories of the reproductive related DEGs are more abundant (Table 1). These include 1) hormone-related DEGs, such as hormone synthesis and degradation enzymes and interacting proteins in relation to JH and Insulin, elevated JH and insulin promotes oogenesis and inhibits immune responses (Galagovsky et al. 2014, Schwenke et al. 2016); 2) egg development-related DEGs, such as *Yolk protein*, *Vitellogenin* and its receptor, which play vital roles in oocyte and embryo development in insects (LeMotte et al. 1989, Van Rompay et al. 2015, Upadhyay et al. 2016); 3) olfactory activity related DEGs, including *Pheromone-binding proteins* and *Odorant receptors*, which play important roles in sexual communication, oviposition, and/or host location by females; and 4) pheromone production-related DEGs, such as *Alcohol-forming fatty acyl-CoA reductase* that function in proportion regulation of each component in the pheromone blend.

From an overview based on gene expression changes from 0 to 24 h postmating and functional annotation (Tables 1 and 2), we suggest that the immune response was largely downregulated shortly after mating (at least from 0 to 6 h postmating), which had some recovery at 24 h postmating. For reproduction, mating incurred response was trivial (few DEGs) shortly after mating (at least till 6 h postmating), but it was largely upregulated (more DEGs and most of them upregulated, particularly in egg maturation related genes that showed much higher LFC, up to 12.73) at 24 h postmating. Considering the fact (Fig. 1) that most of the total DEGs were downregulated during 0 to 6 h postmating (from 51/68 to 214/260) but most of the total DEGs were upregulated at 24 h postmating (816/928), it is possible that there was a trade-off between reproduction and other nonreproductive activities (such as immunity) happened in mated females (Schwenke et al. 2016, Oku et al. 2019); females may shut down nonreproduction activities initially to save limited resources to support the increased energy require in reproduction (egg maturation, host seeking, and oviposition) soon after. *Spodoptera litura* females usually mate only one or two times in their lifetime (Li et al. 2012, Li et al. 2014c). Maintaining a high level of immune activity in *S. litura* may not be necessary or beneficial, given that mating-caused infection should be infrequent due to the low level of polyandry in this insect.

However, the mechanism of mating-induced differential expression in genes related to reproduction and immunity is still unclear and likewise, the role of gene expression changes in the process of mating and postmating physiological/behavioral responses are also unclear. Our previous studies have shown that mating can lead to major changes in reproductive physiology and behavior in *S. litura*, such as being sexually unreceptive and start to lay eggs, accelerated egg maturation and aging process (Li et al. 2012, Li et al. 2014a, Yu et al. 2014, Xu et al. 2019b). Results of the present study suggest that gene expression regulation after mating should be an important internal linkage between mating and postmating behavioral and physiological changes. Previous studies have revealed that both reproductive and immune activities are mediated by hormones (Schwenke et al. 2016, Schwenke and Lazzaro 2017). Generally, increased JH and insulin levels promote oogenesis but inhibit immune responses (Schwenke et al. 2016), while 20E has a positive effect on immune defense by promoting the expression of antimicrobial peptide genes (Meister and Lagueux 2003, Flatt et al. 2008, Zhang and Palli 2009). In *D. melanogaster*, JH synthesis is promoted when the male derived protein Sex Peptide (Acp70A) is transferred to females (Fan et al. 2000). Females mated with wild–type males showed decreased immune response in *D. melanogaster*, whereas females mated with males lacking Sex Peptide were as resistant as virgin females to a bacterial infection (Short et al. 2012). In the present study, we also found obvious expression changes in genes related to hormone synthesis and degradation, and other genes related to hormone function (22 upregulated and nine downregulated; Table 1). These results suggest that endocrine changes likely play important roles in postmating responses in *S. litura*. For example, mating may modify hormone synthesis and degradation in some way, and this change on hormone titers will then mediate postmating responses on gene expression and physiological/behavioral changes. Future studies to clarify the mechanism of mating-induced endocrine regulation and the connection between hormonal changes and altered gene expression will help to archive deeper insights in this field.

## Supplementary Data

Supplementary data are available at *Journal of Insect Science* online.

Fig. S1. GO enrichment of DEGs in Mated-0h vs Virgin-0h group (A), Mated-6h vs Virgin-6h group (B), and Mated-24h vs Virgin-24h group (C). The function of DEGs was divided into three parts: BP (biological process), CC (cell composition), and MF (molecular function). The red bars indicate upregulated DEGs and blue bars indicate downregulated DEGs.

Fig. S2. KEGG pathway enrichment of DEGs in Mated-0h vs Virgin-0h group (A: upregulated DEGs; B: downregulated DEGs), Mated-6h vs Virgin-6h group (C: upregulated DEGs; D: downregulated DEGs), and Mated-24h vs Virgin-24h group (E: upregulated DEGs; F: downregulated DEGs). The size of the dot indicates the number of DEGs in this pathway, and the color of the dot corresponds to different *q*-value ranges.

ieaa003_suppl_Supplementary_Figure_S1

ieaa003_suppl_Supplementary_Figure_S2

ieaa003_suppl_Supplementary_Table_S1

ieaa003_suppl_Supplementary_Table_S2

ieaa003_suppl_Supplementary_Table_S3

ieaa003_suppl_Supplementary_Table_S4

ieaa003_suppl_Supplementary_Table_S5

ieaa003_suppl_Supplementary_Table_S6

ieaa003_suppl_Supplementary_Table_S7

ieaa003_suppl_Supplementary_Table_S8

ieaa003_suppl_Supplementary_Table_S9

ieaa003_suppl_Supplementary_Table_S10

ieaa003_suppl_Supplementary_Table_S11

ieaa003_suppl_Supplementary_Table_S12

ieaa003_suppl_Supplementary_Table_S13

ieaa003_suppl_Supplementary_Table_S14
