# Supplementary figures and images for: Mating-Induced Differential Expression in Genes Related to Reproduction and Immunity in Spodoptera litura (Lepidoptera: Noctuidae) Female Moths
Source: J Insect Sci. 2020 Feb 24;20(1):10. doi: 10.1093/jisesa/ieaa003 (PMC7039226; doi:10.1093/jisesa/ieaa003)

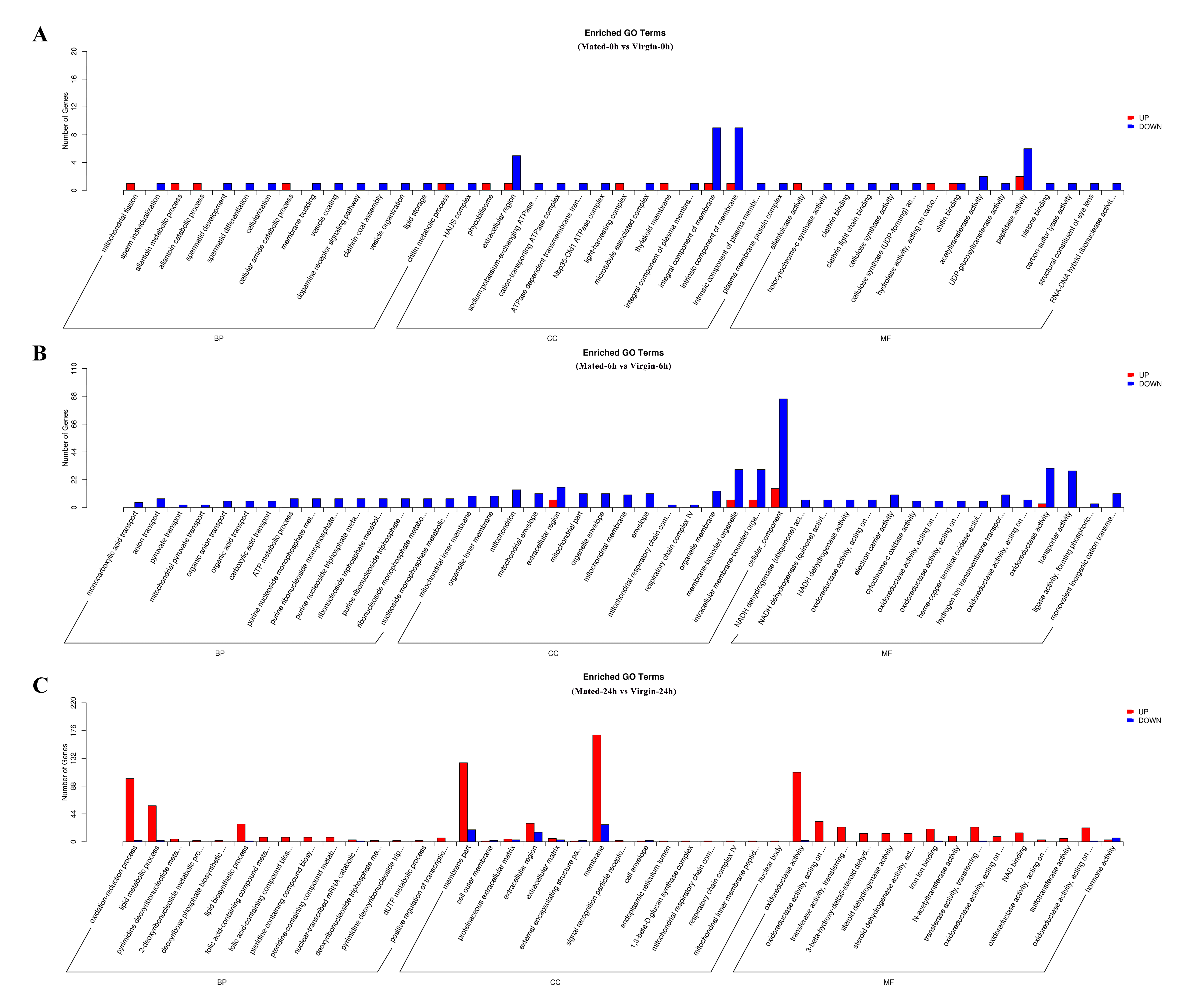

Supplement: ieaa003_suppl_Supplementary_Figure_S1 [file ieaa003_suppl_supplementary_figure_s1.png]

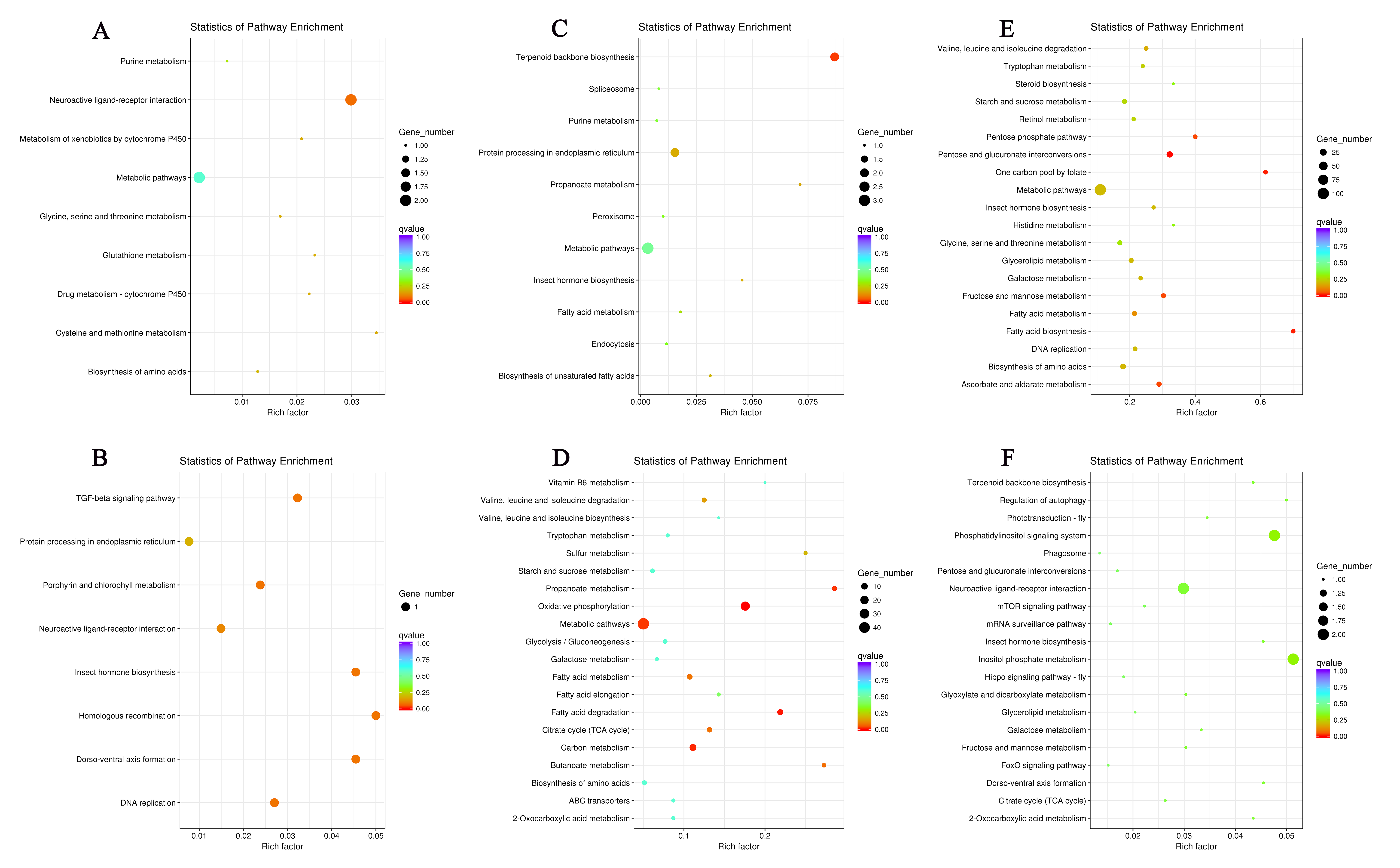

Supplement: ieaa003_suppl_Supplementary_Figure_S2 [file ieaa003_suppl_supplementary_figure_s2.png]
