# Supplementary material for: Mating-Induced Differential Expression in Genes Related to Reproduction and Immunity in Spodoptera litura (Lepidoptera: Noctuidae) Female Moths
Source: J Insect Sci. 2020 Feb 24;20(1):10. doi: 10.1093/jisesa/ieaa003 (PMC7039226; doi:10.1093/jisesa/ieaa003)
Supplement: ieaa003_suppl_Supplementary_Table_S1 [file ieaa003_suppl_supplementary_table_s1.docx]

| **Table S1** Primers for quantitative realtime PCR | | |
| --- | --- | --- |
| Gene ID | Primers | Sequences from 5' to 3' |
| 111357466 | Forward Primer | AGGACGCGACAGCCTCAAAC |
|  | Reverse Primer | GGTGTGTGGGCGATGGAGAC |
| Novel00495 | Forward Primer | GTTCGTGTTCGCGTGTCTGC |
|  | Reverse Primer | TTGGCTGAGCCCAGGACCTC |
| 111357741 | Forward Primer | TCCCATTGAGCGGTTTGACCT |
|  | Reverse Primer | CCGCACCGTCGATAGGGAAT |
| 111350604 | Forward Primer | GCTGACGACTGGCTGCTCAT |
|  | Reverse Primer | TTTGGAGACGTGGCGTCTGG |
| 111358403 | Forward Primer | GTGGCCCTACCTCACAGACG |
|  | Reverse Primer | AGCCAGCAAATCGCCAGTCA |
| 111356125 | Forward Primer | AGCTACCGGAGCCTGTGTCA |
|  | Reverse Primer | GAAGGCCGTGGGATGTCTGG |
| 111355827 | Forward Primer | TGGTGCCGCTTGTCAAGGTT |
|  | Reverse Primer | AATCTCCTCGCCGTCTCCCA |
| 111362176 | Forward Primer | TGTGGCAATTCCTTGGGTCTGG |
|  | Reverse Primer | TCCGACGATGGGAGCCATGA |
| 111351873 | Forward Primer | AGCACAGACATGATTGGCACGA |
|  | Reverse Primer | GGCAGCCAATGCGCAGAAAT |
| 111353785 | Forward Primer | CTGTGCCAGGCAGCTTTACG |
|  | Reverse Primer | ACCACTGGCAGGCACATCTC |
| 111356406 | Forward Primer | CAAGCGGATCTGCCGACTGT |
|  | Reverse Primer | GAAGGACGTCGCCTTTGCCT |
| 111359358 | Forward Primer | AAGGCTGAGGAGGCAGCTCT |
|  | Reverse Primer | GCTGACCCTCTCGTGGATGC |
| 111360320 | Forward Primer | CTGTTACGCCTGCTCAGCCA |
|  | Reverse Primer | ACAGGCGGGCTGCTGTAGTA |
| 111360445 | Forward Primer | CCGCCGCCTAGAGTACAGGA |
|  | Reverse Primer | AGTGCCCAACTCGTGGAAGC |
| 111348786 | Forward Primer | GTTCGTGTGCGCCATGTTGG |
|  | Reverse Primer | GTGGTGATGGTGGCCTGGTG |
| 111351873 | Forward Primer | AGCACAGACATGATTGGCACGA |
|  | Reverse Primer | GGCAGCCAATGCGCAGAAAT |
| 111353785 | Forward Primer | CTGTGCCAGGCAGCTTTACG |
|  | Reverse Primer | ACCACTGGCAGGCACATCTC |
| 111359358 | Forward Primer | AAGGCTGAGGAGGCAGCTCT |
|  | Reverse Primer | GCTGACCCTCTCGTGGATGC |
| 111350990 | Forward Primer | GTGTGCGTCCGGCTCATGTA |
|  | Reverse Primer | ACTCCACGCACTCCCTGTCT |
| 111360733 | Forward Primer | ACTCTGCTCCTCTGCCCACA |
|  | Reverse Primer | GCGGGATATTGCCACTGCCT |
| 111360101 | Forward Primer | ACTGCGTCAGGAGTCTGGGT |
|  | Reverse Primer | AGCGCGGTCCATAGTCAAGC |
| 111363907 | Forward Primer | TCCTCGGGAAACCTCAGCGA |
|  | Reverse Primer | GGCGACCTGGAGACTTGACG |
| Actin | Forward Primer | CAGGCTGTGCTGTCGCTGTA |
|  | Reverse Primer | TAGATGGGCACCGTGTGGGA |
