# Supplementary material for: Mating-Induced Differential Expression in Genes Related to Reproduction and Immunity in Spodoptera litura (Lepidoptera: Noctuidae) Female Moths
Source: J Insect Sci. 2020 Feb 24;20(1):10. doi: 10.1093/jisesa/ieaa003 (PMC7039226; doi:10.1093/jisesa/ieaa003)
Supplement: ieaa003_suppl_Supplementary_Table_S2 [file ieaa003_suppl_supplementary_table_s2.docx]

| **Table S2** Summary of the quality of all sample sequencing data | | | | |  |  |  |  |  |
| --- | --- | --- | --- | --- | --- | --- | --- | --- | --- |
| Sample name | Raw reads | Clean reads | clean bases | Error rate(%) | Q20(%) | Q30(%) | GC content(%) | Total Mapped | Mapped ratio(%) |
| Virgin-0h-1 | 58780158 | 56930902 | 8.54G | 0.03 | 96.46 | 91.12 | 46.74 | 50266416 | 88.29 |
| Virgin-0h-2 | 52018668 | 50574794 | 7.59G | 0.03 | 96.62 | 91.42 | 46.31 | 44968262 | 88.91 |
| Mated-0h-1 | 56273666 | 54889286 | 8.23G | 0.03 | 97.04 | 91.91 | 48 | 49457283 | 90.10 |
| Mated-0h-2 | 47492798 | 46088560 | 6.91G | 0.03 | 97.13 | 92.08 | 47.44 | 41696742 | 90.47 |
| Virgin-6h-1 | 54723354 | 53607950 | 8.04G | 0.03 | 97.14 | 92.09 | 47.37 | 48973010 | 91.35 |
| Virgin-6h-2 | 48418860 | 47465116 | 7.12G | 0.03 | 97.15 | 92.12 | 47.14 | 43171729 | 90.95 |
| Mated-6h-1 | 51065222 | 49788304 | 7.47G | 0.03 | 96.91 | 91.71 | 47.72 | 44470599 | 89.32 |
| Mated-6h-2 | 50216930 | 48796602 | 7.32G | 0.03 | 97.2 | 92.34 | 47.5 | 43821064 | 89.80 |
| Virgin-24h-1 | 54054910 | 52668108 | 7.9G | 0.03 | 96.9 | 91.74 | 47.41 | 46845825 | 88.95 |
| Virgin-24h-2 | 60956416 | 59050024 | 8.86G | 0.03 | 96.99 | 91.85 | 47.97 | 53062697 | 89.86 |
| Mated-24h-1 | 53282028 | 51832584 | 7.77G | 0.03 | 96.61 | 91.09 | 48.13 | 46143032 | 89.02 |
| Mated-24h-2 | 51034866 | 49419560 | 7.41G | 0.03 | 97.59 | 93.46 | 47.96 | 43902567 | 88.84 |
