# Supplementary material for: Mating-Induced Differential Expression in Genes Related to Reproduction and Immunity in Spodoptera litura (Lepidoptera: Noctuidae) Female Moths
Source: J Insect Sci. 2020 Feb 24;20(1):10. doi: 10.1093/jisesa/ieaa003 (PMC7039226; doi:10.1093/jisesa/ieaa003)
Supplement: ieaa003_suppl_Supplementary_Table_S3 [file ieaa003_suppl_supplementary_table_s3.docx]

| **Table S3** Distribution of gene expression levels in different groups by calculating the number of reads for each gene normalized to RPKM | | | | | | | | | | |  |  |
| --- | --- | --- | --- | --- | --- | --- | --- | --- | --- | --- | --- | --- |
| FPKM Interval | Virgin-0h-1 | Virgin-0h-2 | Mated-0h-1 | Mated-0h-2 | Virgin-6h-1 | Virgin-6h-2 | Mated-6h-1 | Mated-6h-2 | Virgin-24h-1 | Virgin-24h-2 | Mated-24h-1 | Mated-24h-2 |
| 0~1 | 7791(42.77%) | 7872(43.21%) | 7963(43.71%) | 7936(43.56%) | 8726(47.90%) | 8351(45.84%) | 8074(44.32%) | 8380(46.00%) | 8166(44.83%) | 8216(45.10%) | 7755(42.57%) | 8157(44.78%) |
| 1~3 | 1585(8.70%) | 1442(7.92%) | 1800(9.88%) | 1532(8.41%) | 1904(10.45%) | 1814(9.96%) | 1754(9.63%) | 1741(9.56%) | 1735(9.52%) | 1788(9.82%) | 1540(8.45%) | 1750(9.61%) |
| 3~15 | 4155(22.81%) | 4012(22.02%) | 4358(23.92%) | 4180(22.95%) | 4098(22.50%) | 4218(23.15%) | 4294(23.57%) | 4160(22.84%) | 4198(23.04%) | 4208(23.10%) | 4099(22.50%) | 4108(22.55%) |
| 15~60 | 3222(17.69%) | 3421(18.78%) | 2742(15.05%) | 3130(17.18%) | 2290(12.57%) | 2550(14.00%) | 2731(14.99%) | 2583(14.18%) | 2715(14.90%) | 2650(14.55%) | 3348(18.38%) | 2785(15.29%) |
| >60 | 1464(8.04%) | 1470(8.07%) | 1354(7.43%) | 1439(7.90%) | 1199(6.58%) | 1284(7.05%) | 1364(7.49%) | 1353(7.43%) | 1403(7.70%) | 1355(7.44%) | 1475(8.10%) | 1417(7.78%) |
