# Supplementary material for: Mating-Induced Differential Expression in Genes Related to Reproduction and Immunity in Spodoptera litura (Lepidoptera: Noctuidae) Female Moths
Source: J Insect Sci. 2020 Feb 24;20(1):10. doi: 10.1093/jisesa/ieaa003 (PMC7039226; doi:10.1093/jisesa/ieaa003)
Supplement: ieaa003_suppl_Supplementary_Table_S4 [file ieaa003_suppl_supplementary_table_s4.docx]

| **Table S4** Pearson's correlation coefficient | | | |  |  |  |  |  |  |  |  |  |
| --- | --- | --- | --- | --- | --- | --- | --- | --- | --- | --- | --- | --- |
| R^2^ | Mated-0h-1 | Mated-0h-2 | Mated-24h-1 | Mated-24h-2 | Mated-6h-1 | Mated-6h-2 | Virgin-0h-1 | Virgin-0h-2 | Virgin-24h-1 | Virgin-24h-2 | Virgin-6h-1 | Virgin-6h-2 |
| Mated-0h-1 | 1 | 0.959 | 0.787 | 0.815 | 0.939 | 0.952 | 0.811 | 0.798 | 0.932 | 0.938 | 0.983 | 0.952 |
| Mated-0h-2 | 0.959 | 1 | 0.918 | 0.927 | 0.92 | 0.885 | 0.956 | 0.939 | 0.904 | 0.938 | 0.902 | 0.951 |
| Mated-24h-1 | 0.787 | 0.918 | 1 | 0.913 | 0.965 | 0.969 | 0.811 | 0.786 | 0.937 | 0.947 | 0.929 | 0.905 |
| Mated-24h-2 | 0.815 | 0.927 | 0.913 | 1 | 0.86 | 0.828 | 0.958 | 0.943 | 0.871 | 0.875 | 0.807 | 0.856 |
| Mated-6h-1 | 0.939 | 0.92 | 0.965 | 0.86 | 1 | 0.965 | 0.851 | 0.826 | 0.967 | 0.981 | 0.951 | 0.959 |
| Mated-6h-2 | 0.952 | 0.885 | 0.969 | 0.828 | 0.965 | 1 | 0.921 | 0.936 | 0.758 | 0.756 | 0.715 | 0.761 |
| Virgin-0h-1 | 0.811 | 0.956 | 0.811 | 0.958 | 0.851 | 0.921 | 1 | 0.983 | 0.91 | 0.913 | 0.922 | 0.945 |
| Virgin-0h-2 | 0.798 | 0.939 | 0.786 | 0.943 | 0.826 | 0.936 | 0.983 | 1 | 0.853 | 0.875 | 0.824 | 0.879 |
| Virgin-24h-1 | 0.932 | 0.904 | 0.937 | 0.871 | 0.967 | 0.758 | 0.91 | 0.853 | 1 | 0.966 | 0.943 | 0.946 |
| Virgin-24h-2 | 0.938 | 0.938 | 0.947 | 0.875 | 0.981 | 0.756 | 0.913 | 0.875 | 0.966 | 1 | 0.868 | 0.919 |
| Virgin-6h-1 | 0.983 | 0.902 | 0.929 | 0.807 | 0.951 | 0.715 | 0.922 | 0.824 | 0.943 | 0.868 | 1 | 0.963 |
| Virgin-6h-2 | 0.952 | 0.951 | 0.905 | 0.856 | 0.959 | 0.761 | 0.945 | 0.879 | 0.946 | 0.919 | 0.963 | 1 |
